# Outcomes in Newly Diagnosed Elderly Glioblastoma Patients after Concomitant Temozolomide Administration and Hypofractionated Radiotherapy

**DOI:** 10.3390/cancers5031177

**Published:** 2013-09-24

**Authors:** Ludovic T. Nguyen, Socheat Touch, Hélène Nehme-Schuster, Delphine Antoni, Sokha Eav, Jean-Baptiste Clavier, Nicolas Bauer, Céline Vigneron, Roland Schott, Pierre Kehrli, Georges Noël

**Affiliations:** 1 Neurology Department, CHU Hautepierre, rue Molière, Strasbourg 67000, France; E-Mail: lnguyen@strasbourg.unicancer.fr; 2 Radiation Oncology University Department, Paul Strauss Center, 3, rue de la Porte de l’Hôpital, BP 42, Strasbourg cedex 67065, France; E-Mails: stouch@strasbourg.unicancer.fr (S.T.); dantoni@strasbourg.unicancer.fr (D.A.); jclavier@strasbourg.unicancer.fr (J.B.C.); nbauer@strasbourg.unicancer.fr (N.B.); cvigneron@strasbourg.unicancer.fr (C.V.); 3 Oncology Geriatric Department, Paul Strauss Center, 3, rue de la Porte de l’Hôpital, BP 42, Strasbourg cedex 67065, France; E-Mail: hnehme@strasbourg.unicancer.fr; 4 Radiation Oncology Department, Soviet-Khmer Friendship Hospital, Pnom-Pehn 12400, Cambodia; E-Mail: sokhaeav@hotmail.com; 5 Oncology Department, Paul Strauss Center, 3, rue de la Porte de l’Hôpital, BP 42, Strasbourg cedex 67065, France; E-Mail: rschott@strasbourg.unicancer.fr; 6 Neurosurgery Department, CHU Hautepierre, rue Molière, Strasbourg 67000, France; E-Mail: pierre.kehrli@chru-strasbourg.fr; 7 Laboratoire EA 3430, Fédération de Médecine Translationnelle de Strasbourg (FMTS), Université de Strasbourg, Strasbourg 67000, France

**Keywords:** hypofractionated radiotherapy, chemotherapy, elderly

## Abstract

This study aimed to analyze the treatment and outcomes of older glioblastoma patients. Forty-four patients older than 70 years of age were referred to the Paul Strauss Center for chemotherapy and radiotherapy. The median age was 75.5 years old (range: 70–84), and the patients included 18 females and 26 males. The median Karnofsky index (KI) was 70%. The Charlson indices varied from 4 to 6. All of the patients underwent surgery. O_6_-methylguanine–DNA methyltransferase (MGMT) methylation status was determined in 25 patients. All of the patients received radiation therapy. Thirty-eight patients adhered to a hypofractionated radiation therapy schedule and six patients to a normofractionated schedule. Neoadjuvant, concomitant and adjuvant chemotherapy regimens were administered to 12, 35 and 20 patients, respectively. At the time of this analysis, 41 patients had died. The median time to relapse was 6.7 months. Twenty-nine patients relapsed, and 10 patients received chemotherapy upon relapse. The median overall survival (OS) was 7.2 months and the one- and two-year OS rates were 32% and 12%, respectively. In a multivariate analysis, only the Karnofsky index was a prognostic factor. Hypofractionated radiotherapy and chemotherapy with temozolomide are feasible and acceptably tolerated in older patients. However, relevant prognostic factors are needed to optimize treatment proposals.

## 1. Introduction

Glioblastoma (GBM) is among the most aggressive tumor types. Its prognosis is associated with a rapidly progressive disease course and a generally fatal outcome. According to the literature, half of all patients diagnosed with glioblastoma are older than 65 years of age. In this population, establishing a standard of care with which to prolong survival without degrading the patient’s quality of life remains very challenging.

Since older patients are often excluded from clinical trials, elderly patients are at risk of receiving inadequate treatment, which could explain the poor outcomes of these patients. Currently, radiation therapy (RT) is recognized to improve survival in elderly patients with malignant gliomas when compared to the administration of only best supportive care [1]. Furthermore, the randomized data do not demonstrate a benefit for the standard 6-week course of RT over a hypofractionated course of RT, given over 2 or 3 weeks [2,3]. Other treatments such as chemotherapy alone have been favorably compared with radiotherapy alone [3,4,5].

The use of concurrent and adjuvant chemotherapy is associated with improvements in patients older than 60 or 65 years of age [6]. However, this treatment modality has been suspected to associate with additional toxicity in older patients, compared to younger patients [7]. A recent retrospective study suggested that no benefit was obtained from the addition of concurrent temozolomide (TMZ), but that a sequential strategy could be more efficient [8]. Therapeutic decisions are increasingly influenced by prognostic factors such as molecular biology [9,10], but such factors do not help physicians to select the best therapeutic option.

We retrospectively analyzed the outcomes of a monocentric elderly population of GBM patients who were treated with hypofractionated radiation in combination or not with concurrent and adjuvant TMZ.

## 2. Patients and Methods

From 09/2005 to 01/2010, 44 patients older than 70 years have been referred to the radiotherapy and oncology departments at the Paul Strauss Center.

## 3. Treatments

All patients underwent surgery. For irradiation, all patients were immobilized with custom thermoplastic masks. Dedicated CT-scans and MRI were performed a maximum of 10 days before initiating irradiation. We did not use preoperative MRI. For all patients, target volume delineation was performed on T1-weighted MRI that had been matched and fused with a CT scan. The gross tumor volume (GTV) was defined as the operative bed plus the contrast enhancement area in the T1-weighted MRI sequence. The clinical target volume (CTV) was designated by the addition of a geometric 2-cm margin that was corrected to the anatomical borders. The planning target volume (PTV) was defined as the CTV plus a 3-mm margin.

Twelve patients received chemotherapy with carmustine wafers and/or temozolomide before radiotherapy. Concomitant chemotherapy was administered to 35 patients, 34 of whom received daily temozolomide at a dose of 75 mg/m^2^ and one who received temozolomide and cilengitide according to a trial protocol. Nine patients did not receive any concomitant chemotherapy because of their general conditions. Of those patients who received concomitant chemotherapy, twenty received adjuvant chemotherapy with a 5-day temozolomide schedule. The temozolomide dose ranged from 150–200 mg/m^2^ per day and was given on five consecutive days per month for a total of at least six months if the patients did not develop complications.

## 4. Follow-Up

On follow-up imaging, tumor progression was defined according to the Macdonald criteria [11]. Individuals who presented with interval clinical deterioration suggestive of tumor progression underwent new, earlier imaging to confirm the diagnoses. At the time of confirmed tumor progression, the patients were treated at the discretion of the referent neuro-oncologist, and the types of administered second-line therapy were recorded.

## 5. Statistical Analysis

### Statistics

The data recorded and included in the analysis were the patient’s age at diagnosis, gender, symptoms prior to diagnosis, symptom duration prior to diagnosis, date and extent of surgery (biopsy, partial or complete resection), MGMT status, pre-radiotherapy steroid requirement, Karnofsky Performance Status before surgery, medical history, tumor site and lateralization. The recorded treatment variables were the type of radiotherapy, radiotherapy parameters, use of a delay between surgery and radiotherapy, use of concomitant and adjuvant chemotherapy, use of antiepileptic drugs, date of radiographic progression, salvage treatment for progression, and date of last known status with the cause of death. MGMT status was determined by the percentage of methylated DNA in a tumor sample (unmethylated if <5%, minor if 5%–30%, methylated if >30% methylated,). Surgery-radiotherapy delays were used to define different groups, which were selected on the basis of quartile distributions.

The survival analysis was conducted according to the Kaplan-Meier method, and the results were compared with the log-rank test. The date of diagnosis used to calculate survival was that of the histopathological examination. The multivariate analysis included the values that were statistically significant in the univariate analysis (*p* < 0.05). For numerical values, comparisons were based on the median as the threshold value. The multivariate analysis was conducted according to the Cox model. All statistical analyses were performed with SPSS statistics v20 (IBM Inc., Armonk, NY, USA).

## 6. Results

### 6.1. Patient Characteristics

We identified 44 elderly (age ≥ 70 years) GBM patients who were treated at the Paul Strauss Center between 2005 and 2010. Their characteristics are summarized in Table 1.

**Table 1:** Patients and treatment characteristics.

*Patient characteristics*	N	%
**Age (years)**		
70–75	18	*41%*
>75	26	*59%*
Median	75,5	
**Gender**		
Male	26	*59%*
Female	18	*41%*
**Pre Radiotherapy KPS**		
<70	12	*27%*
70–100	31	*70%*
undetermined	1	*2%*
Median	70	
**Quality of removal**		
Biopsy	19	*43%*
Partial Resection	14	*32%*
Complete Resection	11	*25%*
**MGMT Status**		
Methylated	12	*27%*
Unmethylated	13	*30%*
Unknown	19	*43%*
**Charlson score**		
4	14	*31.8%*
5	18	*40.9%*
6	5	*11.4%*
7	4	*9.1%*
8	3	*6.8%*
**RPA (according Scott *et al*.)**		
I	17	*38.6%*
II	8	*18.2%*
III	10	*22.7%*
IV	9	*20.5%*
**Preradiotherapy Steroid Requirement**		
Yes	29	*66%*
No	15	*34%*
**Lateralisation**		
Right	19	*43%*
center	24	*55%*
Bilateral	1	*2%*
**Localisation**		
One lobe	30	*68%*
>1 lobe	14	*32%*
**Radiotherapy**		
Hypofractionated	38	*86%*
Standard	6	*14%*
**Concomitant Chemotherapy**		
Temozolomide	34	*77%*
Temozolomide + Cilengitide	1	*2%*
No	9	*21%*
Interruption	7	*21%*
**Adjuvant Temozolomide**		
Yes	22	*50%*
1–3 cycles	7	*32%*
>3 cycles	15	*68%*
No	21	*48%*
with Cilengitide	1	*2%*

The median patient age was 75.5 years old (range, 70–84 years). Twenty-six patients were older than 75 years of age. The median Karnofsky performance status (KPS) was 70% (range, 40–90). The Charlson scores were 4 for 14 patients, 5 for 18 patients and 6 or higher for 12 patients. Additionally, 54% of the patients presented with high blood pressure, and 18% presented with atrial fibrillation. Five patients (11%) had a previous history of cancer (one patient with melanoma, one with prostate cancer and three with breast cancer).

Most patients presented with a combination of symptoms, including monoparesia or hemiparesis (36%), confusion (23%), behavioral changes (18%), seizures (16%) or vision troubles (18%), headaches (14%), memory disturbances (11%), balance troubles (7%), and asthenia (5%). The median duration of symptoms prior to the histological diagnosis was 30 days (range, 2–117 days).

The tumors were localized in the frontal, parietal, temporal, and occipital lobes in 7, 6, 10, and 5 patients, respectively. In 14 cases, the tumors invaded several lobes. The tumors were localized in the right and left hemispheres in 55% and 43% of the cases, respectively. One tumor was bilateral (Table 1).

The patients were classified according to their RPA scores, as described by Scott *et al*. [12]. Specifically, 17, 8, 10 and 9 patients received RPA scores of I, II, III and IV, respectively (Table 1).

### 6.2. Treatment

The treatment characteristics are summarized in Table 1. All patients underwent surgery. Nineteen patients (43%) only underwent biopsy, and most patients underwent tumor resection; 14 patients (32%) underwent subtotal resection and 11 (25%) underwent complete resection. Five patients received implanted carmustine wafers.

All of the patients received radiotherapy. The median delay from histological diagnosis to radiotherapy was 43 days (range, 8–232 days). Thirty-eight patients received a prescribed total dose of 40.5 Gy, given in 15 total 2.7-Gy fractions at a rate of five fractions per week. Six patients received a total radiation dose of 60 Gy, given in 30.2-Gy fractions. The median radiotherapy duration was 22 days (range, 18–51 days). Two patients who were prescribed hypofractionated treatments died before completing radiotherapy and received total doses of 29.5 and 13.5 Gy. Death was considered independent of the radiation procedure.

Concomitant chemotherapy was administered to 35 patients (77%), of whom 34 received daily TMZ at a dose of 75 mg/m^2^ and one received TMZ and cilengitide according to a trial protocol. Nine patients did not receive any concomitant chemotherapy due to their general conditions. Concomitant chemotherapy was interrupted in seven patients due to blood toxicity.

Among all of the patients, twenty-three (52%) received adjuvant chemotherapy with a 5-day TMZ schedule. The average adjuvant TMZ chemotherapy duration was five cycles (range, 1–12). Seven patients received 1–3 cycles of chemotherapy, and 16 received more than three cycles. Twenty-one patients did not receive adjuvant chemotherapy due to their general conditions or because they died prior to treatment. The primary treatments are summarized in Table 1.

At the time of tumor progression, no patients underwent second surgeries, but 10 received additional salvage chemotherapy, which included bevacizumab-irinotecan (n = 7), carboplatine VP16 (n = 2), and carmustine (n = 1) regimens.

### 6.3. Survival and Prognostic Factors

**Figure 1:**
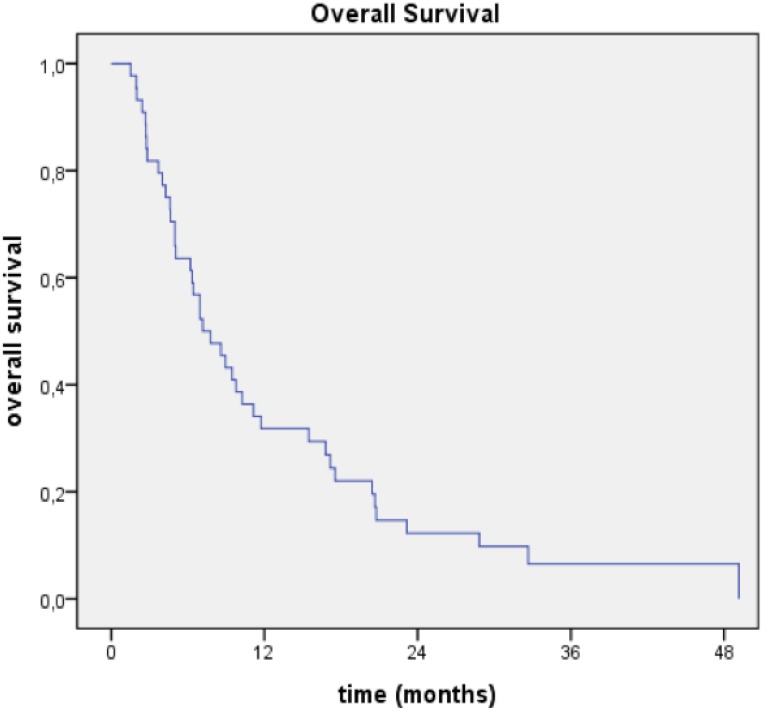
Overall survival for all patients.

At the time of this analysis, 41 of the 44 patients had died. The median overall survival (OS) was 7.2 months. The one- and two-year OS rates were 32% and 12%, respectively. The median progression-free survival was 6.7 months (Figure 1).

**Figure 2:**
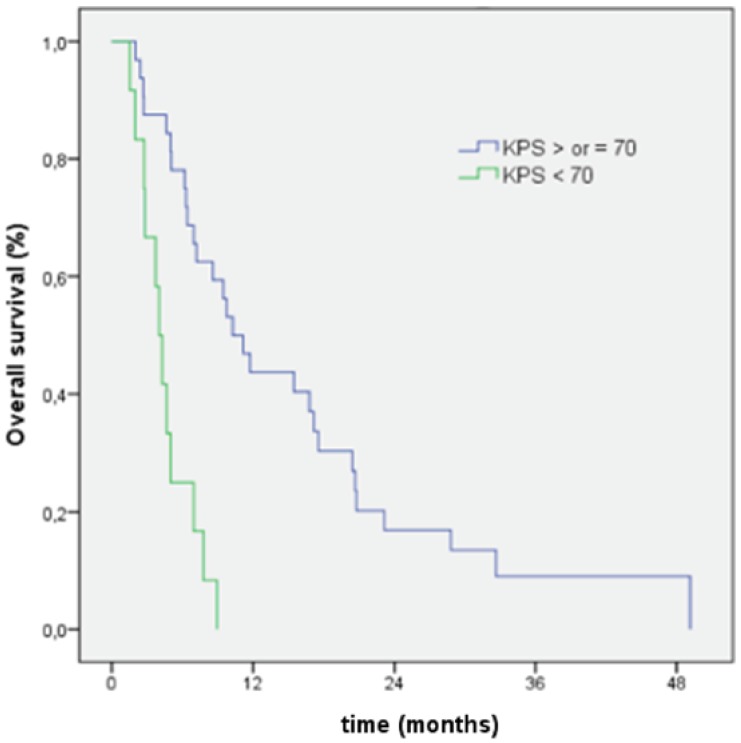
Overall survival curves of patients according to Karnofsky performance score (KPS).

In the univariate analyses, the prognostic factors of OS were KPS (<70 *versus* ≥70; 4.3 *versus* 10.3 months; *p* = 0.0001), concomitant chemotherapy (4 *versus* 9.8 months; *p* = 0.0001), and the number of adjuvant TMZ cycles (1–3 cycles *versus* > 3 cycles, none; *p* = 0.0001). Patient age, gender, the interval between surgery and radiotherapy, surgery extension, radiotherapy schedule, the MGMT status, and the Charlson score were not prognostic factors. In a multivariable analysis, longer overall survival was only associated with KPS (Figure 2).

### 6.4. Charlson Score and Outcome

There were no significant differences between the different Charlson score classes. With a cut-off of 4, we observed median overall survival values of 11.1 months (range, 9.1–20.8) for patients with scores of 4 and 6.9 months (range, 6.8–16.0) for those with scores greater than 4 (*p* = 0.3).

## 7. RPA Score and Outcome

**Figure 3:**
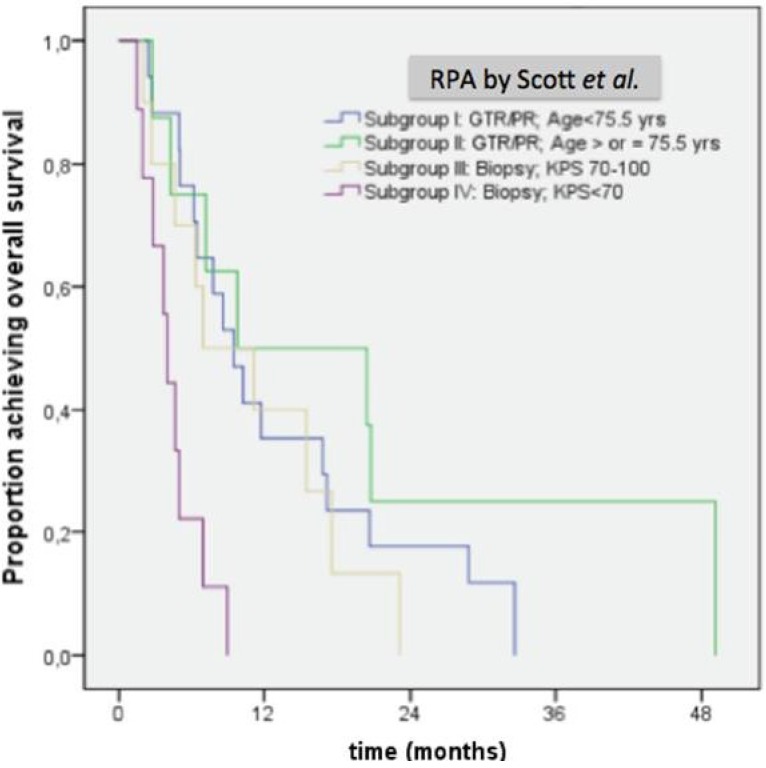
Overall survival of patients according to RPA score (*p* < 0.001). (GTR: gross total resection; KPS: Karnofsky performance score; PR: partial resection).

In an analysis of RPA, the median survival durations for patients with RPA scores of I, II, III and IV were 9.5 months, 9.8 months, 6.9 months and 4 months, respectively (*p* <0.001; Figure 3).

### 7.1. MGMT Methylation and Outcome

**Figure 4:**
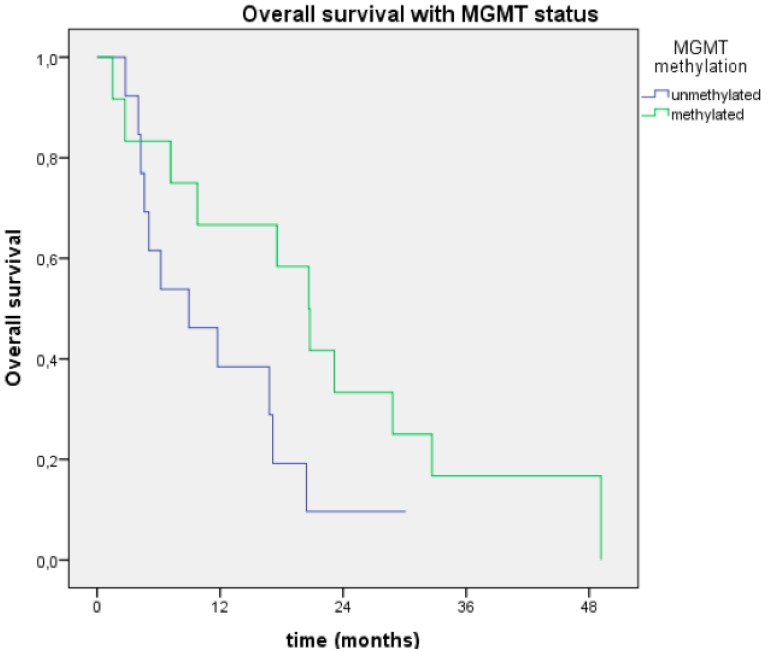
Overall survival curves of patients with methylated or unmethylated MGMT.

MGMT statuses were available in only 24 patients (57%). The MGMT promoter was methylated in 12 (27%) patients and unmethylated in 13 (30%) patients. The median OS among patients with MGMT methylation was 20.6 months (range, 15.2–26.1), compared with 8.9 months (range, 1.1–16) among those without MGMT methylation (*p* = 0.08). The associated one-year OS rates were 64% and 38%, respectively (Figure 4).

### 7.2. Surgery to Radiotherapy Delay

In a univariate analysis, there were no significant differences between the four groups that corresponded to the quartile distribution of delays (*p* = 0.21).

### 7.3. Toxicity

TMZ was generally well tolerated. Concomitant or adjuvant TMZ chemotherapy was definitely interrupted in six patients. The reasons for interruptions included the following: thrombopenia in two cases, cutaneous toxicity in one case, and the patients’ general conditions in three cases. Radiotherapy was not interrupted for medical reasons for those patients who remained alive at the end of radiotherapy.

## 8. Discussion

The superiority of radiotherapy, compared to best supportive care, in elderly patients with KPS >70% has already been demonstrated in a recent randomized trial [1]. Although our series could be considered small in terms of the patient number, we showed an overall survival median comparable to that of a larger series published by Scott *et al*., along with comparable prognostic factors such as the KPS and chemotherapy [13]. In another series, Barker *et al*. found, among other prognostic factors, that combined concomitant RT and CT was a favorable prognostic factor of overall survival [14]. After analyzing the RTOG RPA, the authors concluded that this classification was marginally effective in their series patients. However, in that series, the patients were older than 65 years of age [14]. Other series reported some comparable prognostic factors, but did not always include only patients older than 70 years of age [15,16]. In the largest series of patients older than 70 years of age, Scott *et al*. described a RPA adapted to older patients. This RPA, which was derived from an American series, was implemented in another independent French series with a partial concordance. This RPA remains to be tested in prospective trials [12]. In our series, we showed that the difference in OS according to RPA was significant.

Interestingly, two randomized trials showed that hypofractionated radiotherapy schedules to administer total doses of 40 Gy in 15 consecutive daily fractions or 34 Gy in 15 consecutive daily fractions were well tolerated, with no reductions in survival or quality of life when compared to a normofractionated schedule [2,3]. This result supports the idea that age should not be a limiting factor in glioblastoma treatment [17].

In the present study, we retrospectively analyzed 44 GBM patients older than 70 years of age who had been mainly treated with hypofractionated radiotherapy, with or without concomitant and adjuvant TMZ. The median survival and median progression-free survival were 7.2 months and 6.7 months, respectively. The median survival of this study was comparable to that of other series that evaluated combined hypofractionated radiochemotherapy and adjuvant TMZ (Table 2). However, in some series, the median OS was longer [18,19,20,21,22]. There are multiple explanations for this outcome. The population in our series was older than 70 years of age, while many other series determined a cutoff for elderly people at 60 or 65 years of age, which could influence survival [14]. Recently, Holdhoff *et al*. [23] highlighted the fact that, for patients less than 70 years of age, the standard of care is the schedule used in the EORTC/NCIC trial [24] and neither the Nordic trial nor the NOA-08 included patients in a standard arm [3,25]. Moreover, there was no minimum KPS for the purpose of hypofractionated radiotherapy in this series of patients, which had a minimum KPS of 40%. For patients with KPS >60, the median survival of the patients of our series was 10.3 months, comparable to previous series with patients treated with a combined treatment [6,8,14,20,22], and superior to radiotherapy alone [1,3,7,25] or TMZ alone series [4,5,26].

As demonstrated by the KPS, the MGMT status could help physicians to propose the best treatment schedules for elderly patients with GBM. We showed a trend of significant difference in the overall survival of patients with a methylated MGMT promoter, compared to those with an unmethylated promoter. Previous publications reported comparable results [10,18,21,27]. The Charlson score is also a potential prognostic factor and should be included in all analyses of elderly cancer patients, even those with glioblastoma, as was demonstrated by Fiorentino *et al*. [28].

With regard to the use of TMZ alone as an initial therapy, the results of other studies show survival durations ranging from 6 to 9 months [4,5,26,29,30]. In terms of survival benefit, the recent NOA trial showed that a dose-dense TMZ regimen, given in cycles of 1 week on, 1 week off, is not inferior to radiotherapy alone when treating elderly patients (older than 65 years) with malignant astrocytoma and a KPS of 60 or higher [25]. The Nordic trial concluded that the efficiency of TMZ chemotherapy, administered on five consecutive days every 28 days for up to six cycles or until radiological progression, was comparable to that of hypofractionated irradiation alone in glioblastoma patients older than 60 years of age who had OMS performance statuses of 0 to 2 [3]. Today, conclusions about the use of TMZ alone cannot be fully supported. However, based on the evidence-based results of this prospective study, standard TMZ chemotherapy and hypofractionated radiotherapy are equivalent treatments for elderly patients with high-grade gliomas.

**Table 2:** Results of the literature of elder patients with glioblastoma treated at least with radiotherapy.

Series	Type of trial	Number of patients	Median age (years) IQR	Median KPS (%) IQR	RT total dose dose per fraction	CT	PFS median (months) (IQR) 1-yr PFS (IQR) 2-yr PFS (IQR)	Prognostic factor of PFS	OS median (months) (IQR) 1-yr OS (IQR)/2-yr OS (IQR)	Prognostic factors of OS
Malmström *et al.* [3]	Phase III	93	NA 60–70:51 pts >70:42 pts	NA OMS1-2:78%	No RT	TMZa	NA	NA	8.3 (7.1–9.5) 27% (18–36) NA	Classical irradiation unfavourable
		98	NA 60–70:58 pts >70:40 pts	NA OMS1-2:80%	34 Gy 3.4 Gy	No CT	NA		7.5 (6.5–8.6) 23% (14–31) NA	
		100	NA 60–70:59 pts >70:41 pts	NA OMS1-2:72%	60 Gy 2 Gy	No CT	NA		6.0 (5.1–6.8) 17% (10–24) NA	
Wick *et al.* [25]	Phase III	195	72 (66–84)	70 (20–100)	No RT	TMZ One week on/one week of	3.3 (3.2–4.1) 12% (7.9–17.1) NA	MGMT Extend of resection	8.6 (7.3–10.2) 34.4% (27.6–41.4) NA	MGMT méthylation Extent of resection
		173	71 (66–82)	80 (50–100)	60 Gy 1.8–2 Gy	No CT	4.7 (4.2–5.2) 9.3% (5.5–14.2) NA		9.6 (8.2–10.8) 37.4% (30.1–44.7) NA	
Roa *et al.* [2]	Phase III	47	72.4 ± 5.4 (SD) £	70 60–80	60 Gy 2 Gy	No CT	NA	NA	5.9 44.7% (6 months)	NA
		48	71 ± 5.5 (SD) £	70 60–80	40 Gy 2.67 Gy				6.1 41.7% (6 months)	
Keime Guibert *et al.* [1]	Phase III	75	75 70–84	70 70–100	50 Gy 2 Gy	No CT	14.9 (10.9–22.1)§ NA NA	NA	29.1 (25.4–34.9) § NA NA	NA
		73	73 70–85	70 70–100	No RT	No CT	5.4 (4.4–7.6) § NA NA		16.9 (13.4–21.4) § NA NA	
McAleese *et al.* [31]	Phase II	92	KPS ≤ 50 or KPS 50–90 and age 50–70 or age ≥ 70		30 Gy 5 Gy (3 fractions/week)	No CT	NA	NA	5 12% NA	No factor
Minniti *et al.* [21]	Phase II	71	NA 70–81	70 60–100	40 Gy 2.66 Gy	TMZc TMZc	6 (4.1–8.5) 20% (9–34) 5% (1-12)		12.4 (9.9–15) 58% 20%	KPS Extent of resection MGMT RTOG RPA class
Brandes *et al.* [18]	Retrospective	24	70 65–77	72.5 60–90	59.4 Gy 1.8 Gy	No CT	5.3 (4.8–7.0) 8.3 (2.2–31.4) NA	KPS TMZa	11.2 (9.4–13.3) 31.6 (17.3–57.8) 4.9 (0.6–30.6)	KPS
		32	69 65–74	80 60–90	59.4 Gy 1.8 Gy	PCV	6.9 (5.7–10.6) 15.6 (6.9–35) NA		12.7 (11.2–18.7) 56.2 (41.4–76.4) 6.2 (1.6–23.9)	
		23	68 60–90	77 60–90	59.4 Gy 1.8 Gy	TMZa	10.7 (8.4–16.4) 47.4 (30.7–73.4) NA		14.9 (13.3–24.3) 72.5 (56–94) 20.0 (7.6–53.2)	
Cao *et al.* [8]	Retrospective	57	70 60–86	80 30–100	40 Gy 2.67 Gy	TMZc TMZa	3.9 (2.9–5.3) NA NA	NA	6.9 (4.5–8.6) NA NA	Unfavorable factors: TMZc Limited resection
		55	70 60–81	70 30–100	40 Gy 2.67 Gy	No CT	4.7 (3.2–6.1) NA NA		9.3 (5.9–11.8) NA NA	
Combs *et al.* [20]	Retrospective	43	67 65–76	<70%: 40% pts	60 Gy 2 Gy	TMZc TMZa (5 pts)	4 (0–59) 18% NA	NA	11.0 (2–63) 48% 8%	Extent of resection RTOG RPA class
Glanz *et al.* [30]	Retrospective	54	73.3 70–91	67.4 40–90	60 Gy 1.8 Gy	No CT	NA	NA	4.1 (0.3–22.5) 9.3% NA	KPS
		32	74.5 70–91	67.7 50–90	No RT	TMZa	NA		6.0 (0.7–30) 11.9% NA	
Reyngold *et al.* [22]	Retrospective	31	66 32–90	70–100: 45% of pts	35.5–41.4 Gy 14–15 fractions	TMZc TMZa	NA	NA	11.0 (1–20) NA NA	NA
Iwamoto *et al.* [15]	Retrospective	394	71.9 65–>80	<70%: 24.1%	RT: 80.7% of pts	TMZc:27.2% of pts TMZc or carmustine: 167 pts	NA	NA	8.6 (8–9.4) NA NA	Age KPS Single tumor resection
Scott *et al.* [12]	Retrospective	702	75 70–>83.6	70 <70%:31% of pts	RT: 78% of pts <60 Gy: 54% ≥60 Gy: 46%	CT: 35% of pts	NA	NA	3.1 to 9.3 (1.4–11.2) * NA	RPA $
Scott *et al.* [13]	Retrospective	206	75 70–90	<70%:50% of pts	59,7 Gy (3–70) 2 Gy	CT: 20% of pts TMZa Carmustine carboplatine	NA	NA	4.5 NA NA	KPS Surgical resection RT Chemotherapy
Barker *et al.* [14]	Retrospective	291	71 65–100	80 40–100	NA	TMZc:40% of pts TMZa	NA	NA	12 NA 15% (11–20)	Age RTOG RPA Extent of surgery TMZc
Minniti G. [10]	Retrospective	32	73.6 70–79	80 70–100	60 Gy 2 Gy	TMZc TMZa	7 (5–9) 16% (4–28) NA	NA	10.6 (8.6–12.6) 37% (23–50) NA	KPS
Sijben *et al.* [7]	Retrospective	19	67 64–74	80 60–90	60 Gy 2 Gy	CT:19 pts TMZa TMZc	6 (1.6–24.7)	NA	8,5 (2–24.7) NA NA	Extent of resection KPS TMZa-TMZc
		20	70 65–82	70 50–90	45 Gy 2.66 Gy	No CT	4.1 (1.5–14.2)	NA	5.2 (1.5–14.2) NA NA	
Bauman *et al.* [32]	Retrospective	29	≥65	≤50–100	30 Gy 3 Gy	No CT	NA	NA	6 NA NA	NA
Villa *et al.* [33]	Retrospective	91	>70:47% of pts	<70%:52% of pts	RT: 50% of pts 54–66 Gy 1.8–2 Gy or 1,5 Gy × 2/day	CT:10 pts Carmustine PCV	NA	NA	4.5 NA NA	RT
Mohan *et al.* [34]	Retrospective	102	74.5 70–87	70.5	RT: 77 pts >55 Gy 1.8–2 Gy: 58 pts <40 Gy 3 Gy:19 pts	Carmustine PCV Carboplatine	NA	NA	5.3 (0.1–36.9) NA NA	RT RTOG RPA
Patwardhan *et al.* [35]	Retrospective	30	>59	67.9 ± 2.8 (SD)	RT for 15 pts 48–64 Gy 2 Gy	BCNU TMZa	NA	NA	3.2 13–6 according to treatment	Treatment
Pierga *et al.* [36]	Retrospective	30	73 70–79	66 30–100	45 Gy 1.8 Gy	BCNU	26 § NA NA	NA	36 (8–70) § NA NA	NA
Hoegler and Davey [37]	Retrospective	22	73 70–78	70.4 30–90	37.5 Gy 2.5 Gy	No CT	NA	NA	8 (4.8–9.6) NA NA	KPS
Present series	Retrospective	44	75.5 70–84	70 40–90	40.5 Gy 2,7 Gy	TMZc TMZa	6.7 (4.3–9.1) 35% 9%	NA	7.2 (4.4–49.1) 32% 12%	KPS TMZa

BED: Biologically Equivalent Dose; CT: chemotherapy; IQR: Interquartile Range; KPS: Karnovski Performance Status; NA: non available; OS: overall survival; PCV: procarbazine, lomustine, vincristine; PFS progressive survival; pts: patients; RT: radiotherapy; SD: standart deviation; TMZa: Temozolomide adjuvant or adjuvant-like (monthly treatment); TMZc: temozolomide conconcomitant; *: according to RPA; $: design from the series; £: mean (not median); weeks (not months).

Due to its ease of delivery, TMZ alone could be considered useful mainly for patients with the worst performance statuses. However, in this population with highly altered general conditions, Gallego *et al.* underlined the feasibility of TMZ alone in elderly patients with KPS < 70. Hematologic and cutaneous toxicities were the principal deterrents of this approach [29]. These complications were also reported in the NOA-08 trial [25]. While Minniti *et al*. described similar complications, the authors also noted that the most common adverse event was grade 2 or 3 fatigue, which occurred in 10% of patients during RT and in 17% of patients during adjuvant TMZ chemotherapy. In contrast to hematologic toxicity, which is often asymptomatic, fatigue can have a substantial impact on a patient’s quality of life [10].

Wick *et al.*, who concluded that the major finding of the NOA-08 trial was the strong predictive power of the MGMT promoter methylation status for event-free survival, determined another point that could facilitate decisions about more relevant treatments. MGMT promoter-methylated tumors responded better to TMZ than to radiotherapy, whereas the opposite was true for unmethylated tumors [25].

For elderly patients, the quality of life is a major concern in radiation trials, and randomized trials have evaluated patients’ quality of life [2,3,5]. These trials showed that radiation therapy was not deleterious to the quality of life [1,2], even if patients in the Nordic trial who received TMZ reported a better quality of life than those who received radiotherapy [3]. Furthermore, these studies showed that there was no quality of life reduction due to the use of high-dose irradiation fractions *versus* a normofractionated schedule [2,3]. However, the effect of the time interval between surgery and the beginning of radiotherapy on the quality of life remains uncertain. Longer waiting times can induce anxiety in both patients and physicians, who may be concerned about tumor progression before initiating treatment. However, some previous large series did not find any differences in the quality of life, according to this time interval [38,39,40]. In this series, we did not find any difference in survival with respect to different time intervals. Do *et al.* found that patients with one or more of the following factors were treated earlier than patients without these factors: increased age, biopsy surgery only, and lower performance status [39]. This finding could partially explain the lack of differences observed in our series.

The potential toxicity associated with combined chemoradiation in elderly patients leads many physicians to choose less aggressive treatments. One of the weaknesses of our study is the insufficient data regarding the adverse effects of combined therapy, particularly neurocognitive effects and quality of life follow-ups. Many previous retrospective studies did not conduct formal neurocognitive testing. Standardized assessment scales of essential domains such as daily living activities, communication, cognitive function and memory, depression, and quality of life would provide necessary information about the uses of any approach in this patient population. The quantification of such complications remains challenging, since modifications in neurocognitive status are known to be often attributed to epilepsy, antiepileptic drugs, and disease progression. In a prospective series of elderly patients who were treated with a combination therapy, Brandes *et al*. suggested that the median time to the development of neurologic toxicity was 6 months, whereas the time to progression was 9.5 months [18].

However, some questions remain unresolved. In particular, what age should be used to define someone as elderly? The cut-off age is controversial; Wick *et al*. regarded patients older than 65 years of age as elderly [25], but others have defined elderly people as those older than 70 or 75 years of age [28,41]. Another issue regards the heterogeneity of the elderly population. Comorbidity analyses have become much more important and could have predictive roles in survival. Even if we cannot demonstrate this hypothesis in our series, the comorbidity status could be a useful way to select elderly patients.

## 9. Conclusions

Our results indicate that a combined treatment regimen is tolerable and has few recorded adverse effects. The establishment of prognostic factors for elderly people remains challenging. Ultimately, the best treatments could be efficiently proposed according to these factors.
